# Pulmonary artery stenosis caused by a large aortic arch pseudoaneurysm detected 10 years after a minor trauma

**DOI:** 10.15171/jcvtr.2016.09

**Published:** 2016-03-15

**Authors:** Jalal Zamani, Kamran Aghasadeghi, Khalil Zarrabi, Alireza Abdi Ardekani, Abdolali Zolghadrasli

**Affiliations:** ^1^Department of Cardiology, School of Medicine, Shiraz University of Medical Sciences, Shiraz, Iran; ^2^Cardiovascular Research Center, Shiraz University of Medical Sciences, Shiraz, Iran; ^3^Department of Cardiac Surgery, School of Medicine, Shiraz University of Medical Sciences, Shiraz, Iran

**Keywords:** Aortic Arch Pseudoaneurysm, Pulmonary Artery Compression, Blunt Chest Trauma

## Abstract

Pseudoaneurysm of aorta is a rare condition usually seen after aortic surgeries or serious accidents. Here we report a 60 years old man without any previous medical condition who presented with non-specific symptoms and underwent different investigations for more than 1 year, until the presence of a continuous murmur raised suspicion toward his cardiovascular system. In echocardiographic and computed tomography (CT) angiographic studies a large pseudoaneurysm of aortic arch with compression effect on pulmonary artery was detected. At this stage he remembered having suffered a minor trauma 10 years ago. He finally underwent operation and his aortic wall was repaired successfully with a patch. This case highlights the importance of thorough history taking and physical examination in patients irrespective of symptoms and high index of suspicion to detect this life-threatening condition.

## Introduction

Stenosis of the right ventricular outflow tract due to extrinsic compression has been described in patients with mediastinal tumor, pericardial cyst and vascular abnormalities like aneurysms.^1^ Pseudoaneurysm of the aorta is a rare but potentially life-threatening condition which is usually encountered after surgeries of the aortic valve and blunt trauma to the chest.^2-4^ Unusual etiologies include infection, connective tissue diseases, vasculitis and spontaneous formation.^3,5,6^ Here we present a case who presented with non-specific symptoms and underwent extensive investigation until he was finally diagnosed with a large pseudoaneurysm of aortic arch and underwent successful operation.

## Case Presentation

The patient was a 60-year-old man who referred to our emergency department due to worsening dyspnea and hemoptysis since 2-3 days prior to admission. He reported to have dyspnea and hoarseness during the previous year. He had undergone a direct laryngoscopy which had revealed left vocal cord palsy and a chest computed tomography (CT) scan which had shown a mediastinal mass and with possibility of a malignancy process a direct needle biopsy was done which demonstrated inflammatory cells in the background of blood. His past medical history only included a mild stroke 6 years before without any sequel.

At the emergency department he had pulse rate of 86 beats/min, respiratory rate of 18/min and blood pressure of 135/80 mm Hg. He had a continuous murmur in left sternal and pulmonic area and decreased breathing sound in left hemithorax.

**Figure 1:**
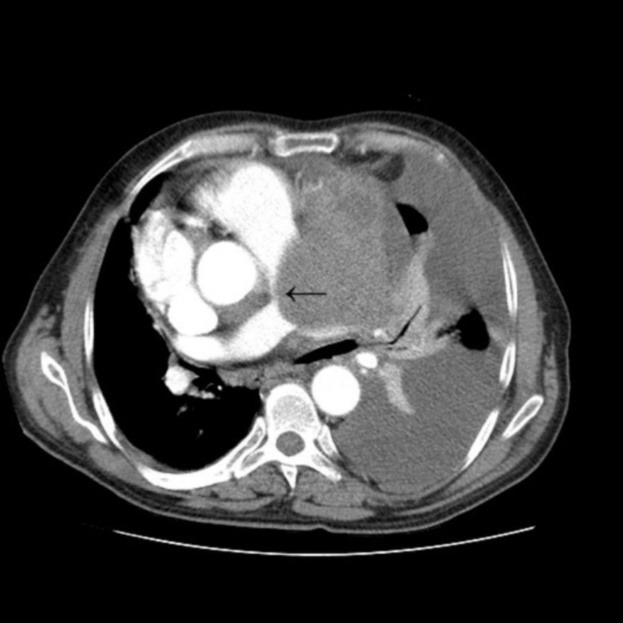
Chest CT scan with IV contrast. Black arrow pointing to pressure effect of hematoma on main and left pulmonary artery.

**Figure 2:**
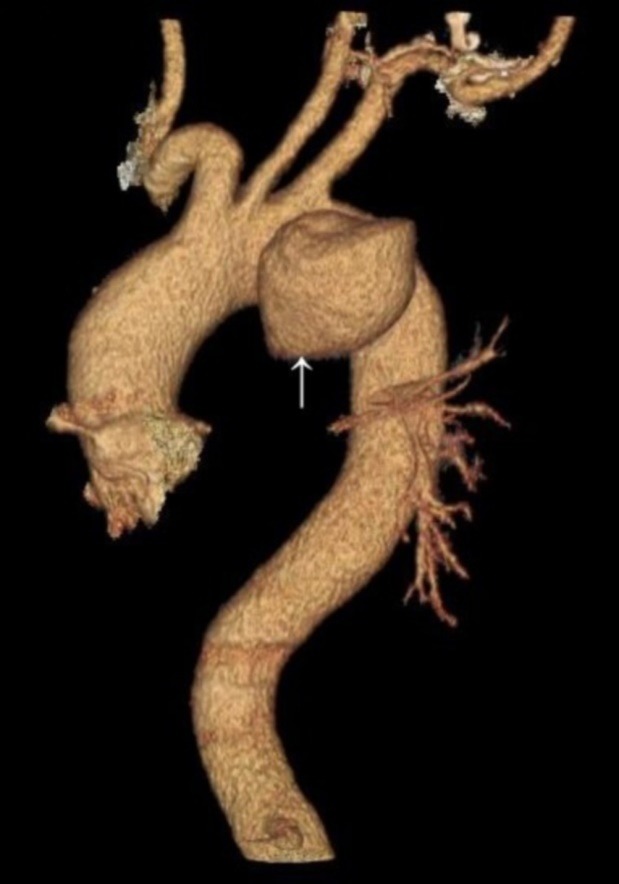
CT angiography of aorta. White arrow pointing to pseudoaneurysm of aorta distal to subclavian origin.

A chest x-ray was obtained which showed a large density in mediastinum and left sided pleural effusion. In echocardiography he had normal left ventricle but dilated right sided chambers with supravalvular pulmonic stenosis. Trans-esophageal echocardiography revealed a pseudoaneurysm of distal aortic arch with clot formation and compression of the main and left pulmonary artery. As a result a chest CT scan with contrast and angiographic construction was done which confirmed the previous finding of a 45 mm × 37 mm pseudoaneurysm in lateral side of aortic arch distal to left subclavian origin with associated 9.5 cm × 10 cm hematoma in mediastinum (Figure 1 and 2). At this stage and upon further investigation he remembered to have fallen down from a few stairs 10 years ago which did not result in any injuries and accordingly he did not seek medical advice.

The patient initially refused operation but a few days later developed severe chest pain and so finally agreed to a surgical plan.

**Figure 3:**
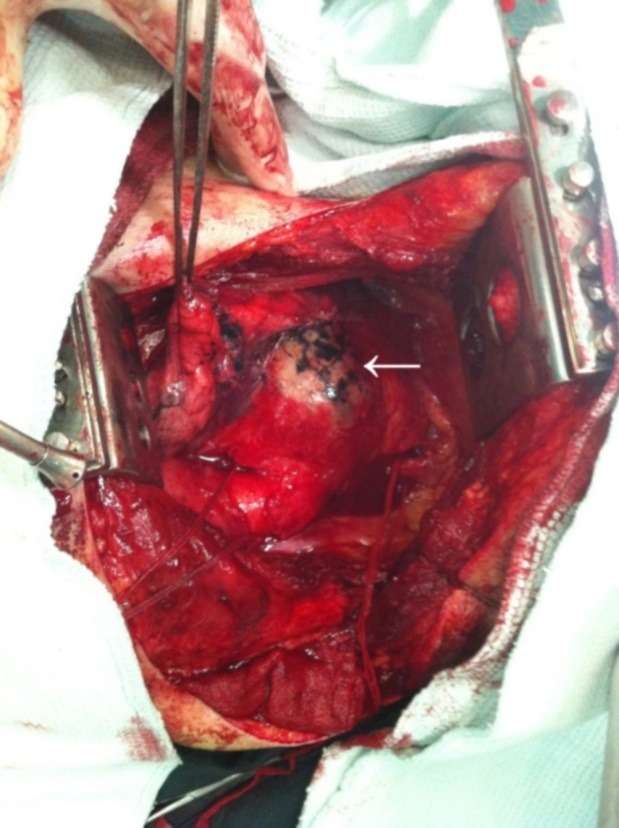
The Pseudoaneurysm at surgery before evacuation of mediastinal hematoma.

The patient had a preoperative selective coronary angiography via right radial artery which was normal. He underwent left thoracotomy via femorofemoral bypass. A large pseudoaneurysm of distal aortic arch was detected (Figure 3). Large hematoma was evacuated from the mediastinum and the aortic wall was repaired with a patch.

His hospital course in intensive care unit (ICU) was uneventful and was discharged after 1 week.

## Discussion

Most cases of aortic pseudoaneurysm are reported after surgery especially those involving the aortic valve with predisposing factors including graft infection, dissection of native aorta and tissue necrosis due to overuse of biologic glue.^2-5^ The second most common cause of aortic pseudoaneurysm is blunt trauma to the chest.^2,3^ These usually occur after deceleration injury, are usually accompanied with significant injuries to other organs and are mostly fatal.^7-9^ The main context is in high speed automobile crashes (>100 km/h) with a deceleration change in velocity of more than 32 km/h.^8^ Most injuries to the aorta occurs with head on impact with side and rear impact occurring much less often.^8^ Other causes of blunt trauma which result in pseudoaneurysm include motorcycle and aircraft crashes, auto-pedestrian collisions, falls and crush under weight injury.^9^ In addition to the aorta, other vascular injuries can occur with the innominate, subclavian and left common carotid arteries accounting for almost all of the injuries.^8^ Pulmonary vessels, azygos vein and caval vessel injuries are very rare in this setting.^8^ Among those who survive, in about 2%-5% the diagnosis of aortic injury is missed and is later discovered as chronic pseudoaneurysm.^10^ If left untreated, chronic pseudoaneurysms typically expand progressively, compress the adjacent structures, are source of infection and systemic embolism and finally rupture.^5,10^ Usually chronic pseudoaneurysm does not produce significant symptoms and is detected accidentally when imaging is done for other reasons, however it may be associated with various symptoms including chest pain, syncope, hoarseness, dyspnea and cough due to airway compression, hemoptysis due to either aortobronchial fistula or erosion into lung parenchyma and finally external pressure on pulmonary artery manifested by a continuous murmur.^1,3,5,10^ Our patient demonstrated hoarseness and dyspnea and finally hemoptysis most probably due to erosion into lung parenchyma as no fistula was reported either in the CT scan or in the operation room by the surgeon.

Chronic pseudoaneurysm may mimic some other pathologies of aorta including penetrating atherosclerotic ulcers, infected mycotic saccular aneurysm and vasculitis. Traumatic pseudoaneurysms are usually located at the isthmus, although they may occur anywhere in the aortic tree, and can be distinguished from penetrating ulcers from lack of dilation of adjacent aortic segments and lack of significant atherosclerosis as was the case for our patient.^9,10^ Similarly, infected aneurysm and vasculitis are not commonly found near the isthmus and are associated with lobulations and inflammation in aortic wall.^9^ In any case, CT angiography seems to be the best modality to confirm the diagnosis.^2,8,10^ Our patient had a history of falling from a few stairs 10 years earlier which was not severe enough for him to seek medical attention. Whether this has caused the pseudoaneurysm is a subject of debate. As noticed most traumatic injuries are associated with severe accidents while our patient had a minor trauma, but the location of the pathology and lack of atherosclerosis and other pathologies elsewhere in the aortic wall point to a traumatic origin. There is a possibility of spontaneous pseudoaneurysm, but most patients had hypertension as risk factor and presented acutely to the hospital with chest pain or back pain which was not the case for our patient.^6^ So this patient is interesting in this aspect that it may be the first reported case in literature for the delayed presentation of pseudoaneurysm only after a minor injury and blunt traumatic injury to aorta should be suspected even after an insignificant chest trauma with the deceleration injury mechanism.

There is one intriguing point in this case; our patient had non-specific symptoms for about 1 year. Different invasive and noninvasive investigations were done for him which not only yielded futile results but also were misleading in the sense that a malignancy process was suspected and needle biopsy was done and if it was not for the pickup of the continuous murmur during physical examination, there was possibility of further delays to diagnosis. This case is another evidence that complete physical examination in any patient referring with non-specific complaints is a necessity and imaging studies cannot replace a thorough history taking and physical examination. This case highlights that a high index of suspicion is needed to diagnose this critical condition and even when the best imaging techniques are available without the proper diagnostic algorithm in mind, there may be no satisfactory results.

## Ethical Issues

The patient gave informed consent to the use of his medical data for publication.

## Competing Interests

None
